# State Health Officer of Texas

**Published:** 1898-09

**Authors:** 


STATE HEALTH OFFICER OF TEXAS.—Upon the death of the lamented Dr. Swearingen, the governor appointed Dr. Walter

F. Blunt, of Lockhart, to the office of State Health Officer and he at once entered on the discharge of his duties. Dr. Blunt is a native of Virginia, a graduate of the Texas Medical College of the class of 1868, and is sixty-three years of age. He has had a large experience in yellow fever and for many years served under Dr. Swearingen as quarantine officer of the post of Galveston, a most difficult and responsible position. That he was most efficient in that position is evidenced by his re-appointment under several administrations. That Dr. Blunt will make an equally efficient officer in this wider field goes without saying. He is a pleasant, courteous, gentleman, and has a wide circle of friends who are rejoiced at his promotion to so distinguished a position.

---

"You aint good lookin' and you can't come in," Mr. Homeopath; there is an *examination* in the way, and *homo's* are as much afraid of an examination as the devil is of "holy water." I see that "Senator Allen, of Nebraska," has introduced a resolution in Congress "forbidding discrimination against any school of medicine" (?) in appointments in the army and navy.

That's all right; let Mr. McKinley appoint every mother's son of the "homos." It doesn't "go" unless a board of examiners says so; and if they can *stand the examination* that is all that is demanded. They don't dare to try it.

---

Dr. A. W. Fly, the plucky mayor of Galveston, on August 29th, placed himself at the head of the police force, and, pistol in hand, quelled a serious labor riot. I am going to nominate Fly for secretary of war, vice the lame duck now holding down that office.

